# Mechanisms of aging in the cardiovascular system: challenges and opportunities

**DOI:** 10.3389/fimmu.2025.1635736

**Published:** 2025-11-05

**Authors:** Xiaoying Zhao, Xinyue Yang, Yumeng Lin, Ruolan Lei, Wei Ding, Xinying He, Ying Cao, Dechou Zhang, Ping Liu, Meirou Liang, Zhongyu Han, Yu Jiang

**Affiliations:** ^1^ Department of Gerontology, The Affiliated Traditional Chinese Medicine Hospital, Southwest Medical University, Luzhou, China; ^2^ Health Management Center, Nanjing Tongren Hospital, School of Medicine, Southeast University, Nanjing, China; ^3^ Department of Nutrition, The First People’s Hospital of Zigong, Zigong, China; ^4^ The First Affiliated Hospital of Jinzhou Medical University, Jinzhou, China; ^5^ The First Clinical Medical College of Shaanxi University of Chinese Medicine, Xi’an, China; ^6^ Zhongda Hospital, School of Medicine, Southeast University, Nanjing, China; ^7^ College of Traditional Chinese Medicine, Chongqing College of Traditional Chinese Medicine, Chongqing, China

**Keywords:** cardiac microenvironment, aging, cardiac aging, therapy, inflammation

## Abstract

Cardiovascular diseases (CVDs) pose a significant threat to the health of the elderly population. As the global population ages and medical management remains imperfect, reducing the medical burden of CVDs is of great importance. Aging is a complex process that contributes to the development and progression of CVDs through various mechanisms. The manuscript reviews the mechanisms of aging and their impact on the cardiovascular system. We explore the role of aging in the cardiac microenvironment, highlighting the changes that occur in the heart’s cellular and molecular landscape as a result of the aging process.

## 1 Introduction

**Figure 1:**
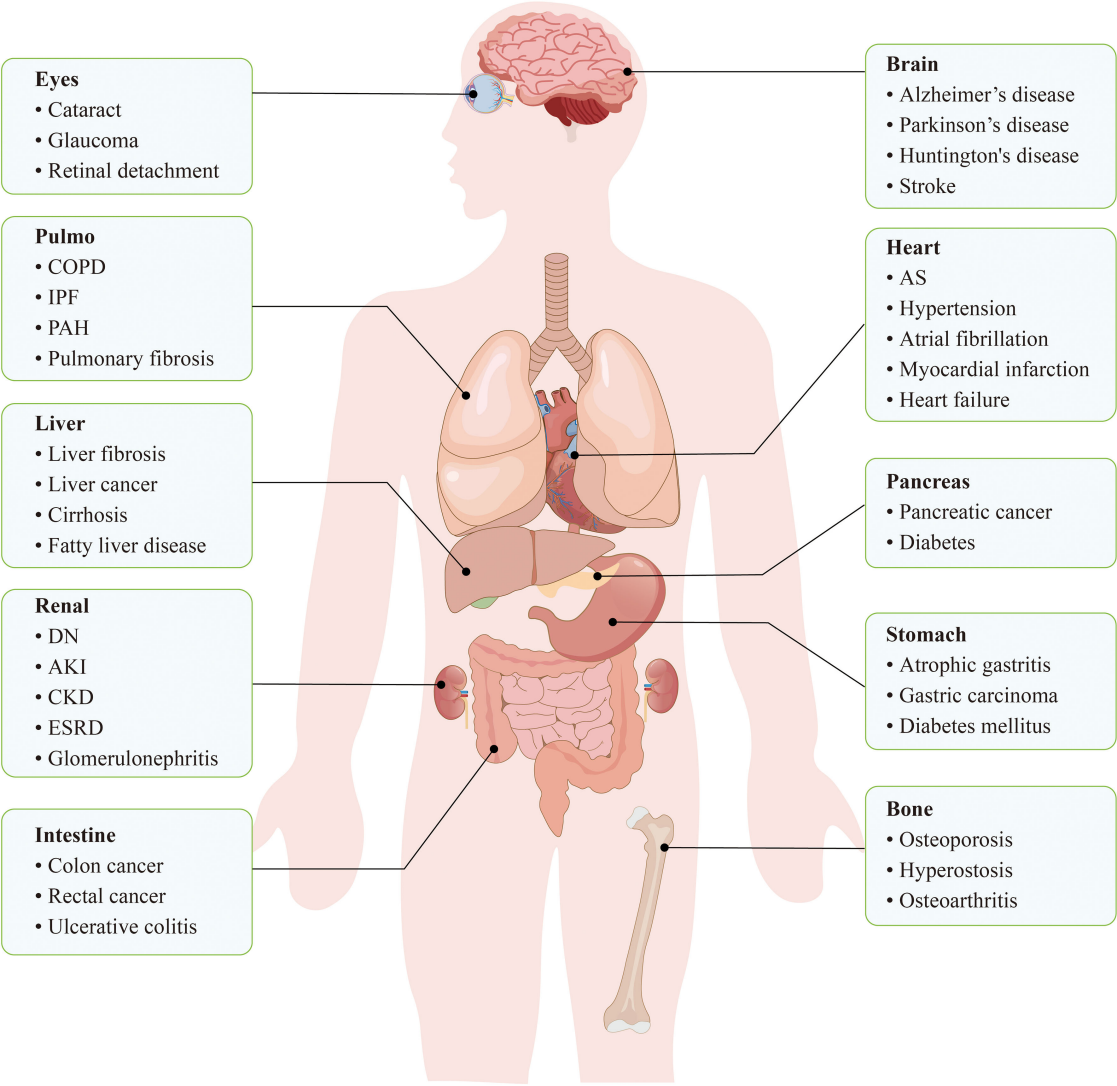
Cellular senescence is closely related to a variety of diseases throughout the body. In the cardiovascular system, cellular senescence leads to dysfunction of ECs, VSMCs, etc., which in turn increases the risk of diseases such as hypertension, AS, and cardiac infarction. In the nervous system, neuronal senescence may trigger neurodegenerative diseases, such as Alzheimer’s disease and Parkinson ‘s disease. In conclusion, cellular senescence accumulates in systemic tissues and becomes a potential trigger for a variety of diseases.

Enhanced living conditions and medical advancements have significantly prolong human lifespan (1). The proportion of the population aged 65 years and over is predicted to increase substantially worldwide by 2030, accounting for approximately 19% of the total population (2). Old age is generally regarded as a major and nonmodifiable risk factor for chronic, life-threatening conditions (3), including CVDs, cancer (4, 5), and neurodegenerative diseases (6) (**Figure 1**). Among these, CVDs represent the leading cause of mortality among the elderly (7). During body ageing, the accumulation of senescent cells may adversely affect tissue homeostasis (8, 9). Therefore, reducing the accumulation of senescent cells is important for slowing the onset and progression of ageing-related CVDs.

In the cardiac environment, aging emerges as a stress response triggered by numerous stimuli, such as telomere attrition, virus infection, hypoxia, oxidative stress, mitochondrial dysfunction, protein imbalance, and impaired autophagy (10). Increasing evidence illustrates the complex associations between cardiovascular cellular senescence and the pathogenesis as well as progression of CVDs, including atherosclerosis (AS), arterial stiffening, aortic aneurysms, myocardial fibrosis and heart failure (11).

**Figure 2:**
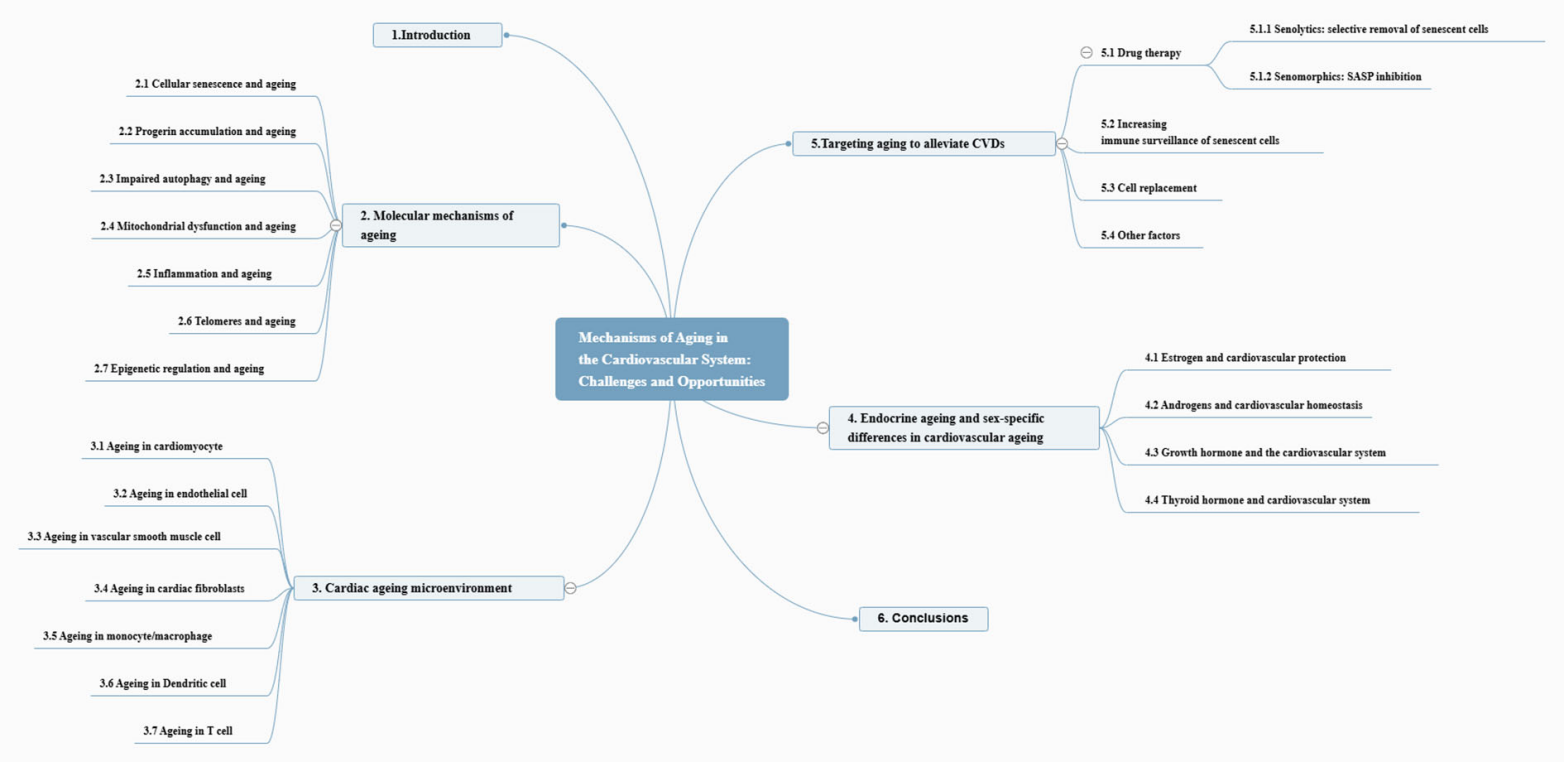
Overview of this review.

This manuscript discusses the phenotypic expressions and underlying molecular pathways correlated with the ageing process and their contribution to the development of CVDs. Additionally, we assessed the advantages and challenges of targeting senescent cells in preventing and managing ageing-related CVDs. The entire framework of the article is depicted in **Figure 2**.

## 2 Molecular mechanisms of ageing

**Figure 3:**
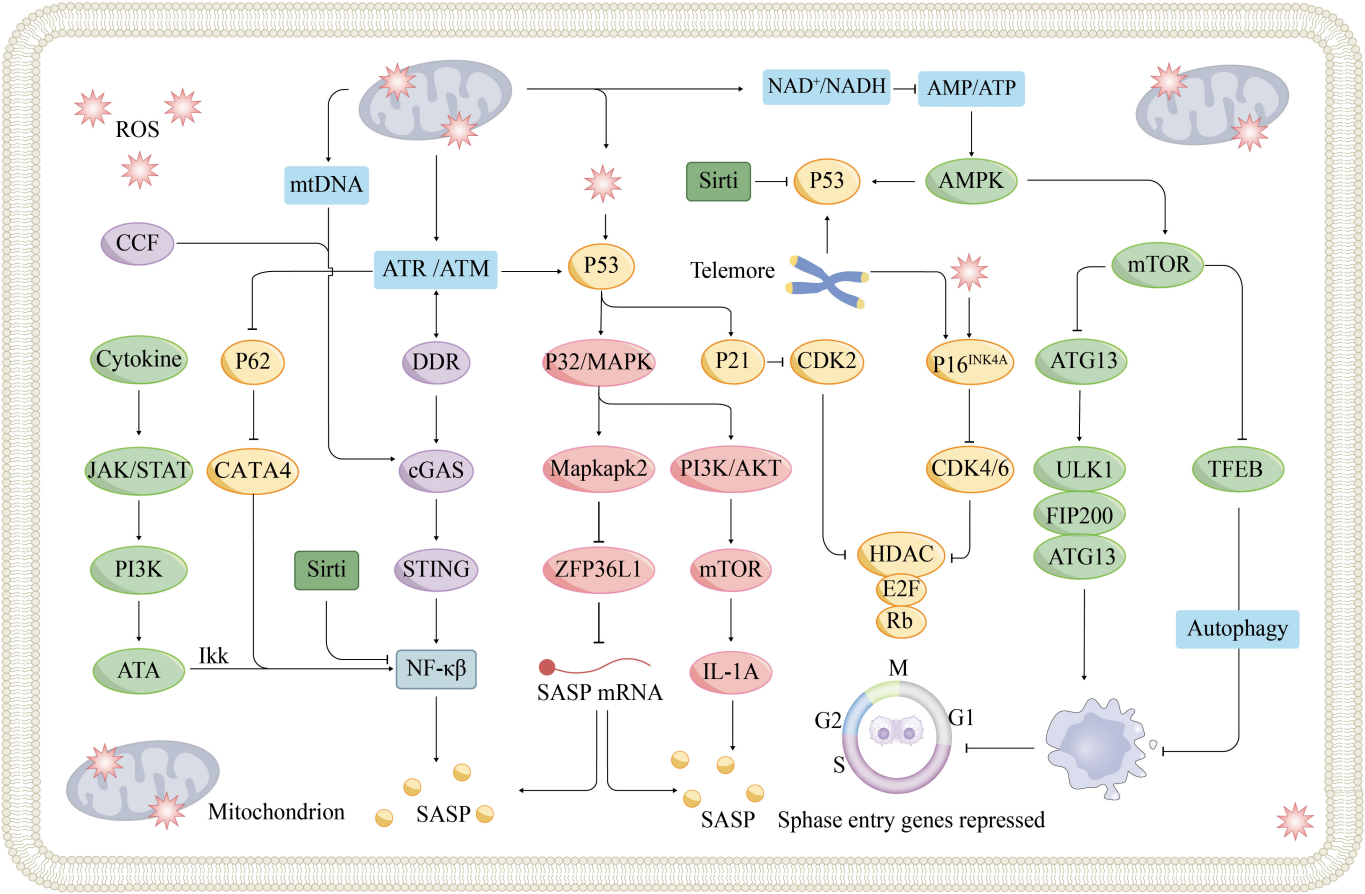
Signaling pathways and mechanisms of cellular senescence. Cellular senescence is a cellular state triggered by stress injury and some physiological processes, which is mainly divided into replicative senescence and non-replicative senescence. Replicative senescence is caused by telomere shortening due to continuous division of normal cells, and when telomeric DNA shortens to a certain extent, cells automatically turn on the ageing program and prevent cell cycle progression through the p53/p21 and p16^INK4A^/Rb signaling pathways. Non-replicative ageing can be triggered by various stress factors, and nuclear DNA damage is an important role. DNA damage-activated DDR can lead to p53 activation via ATM or ATR kinase activation, which in turn triggers cell cycle arrest. DDR can also induce SASP secretion via the cGAS-STING pathway. SASP components secreted by nearby senescent cells, such as IL-6, trigger the JAK-STAT signaling pathway, the so-called paracrine-induced senescence. In addition, mitochondrial dysfunction has been implicated as a driver of cellular senescence, mainly through three different mechanisms, which have been described in detail previously. ATM, Ataxia-Telangiectasia mutated; ATR, Ataxia-Telangiectasia and Rad3-related protein; CCF, cytoplasmic chromatin fragments; MAPK, mitogen-activated protein kinases; mtDNA, Mitochondrial DNA; ULK1, UNC-51-like kinases 1.

The ageing process in the heart is marked by several key molecular mechanisms (**Figure 3**). Aging hinders tissue regeneration, whereas the accumulation of progerin disrupts nuclear function. Impaired autophagy and mTOR pathway dysfunction lead to the accumulation of damaged cellular components. Mitochondrial issues impact energy metabolism and contribute to oxidative stress. When dysregulated, the cGAS-STING signaling pathway can trigger inflammation. Telomeres shorten with age, triggering a DNA damage response (DDR) that can lead to senescence. The senescence-associated secretory phenotype results in the release of inflammatory factors that degrade the surrounding tissue. Epigenetic changes also influence the expression of genes related to cardiac ageing. Together, these factors contribute to the ageing characteristics of the heart.

### 2.1 Cellular senescence and ageing

Cellular senescence, characterized by irreversible exit from the cell cycle and entry into a state of growth arrest, was initially proposed by Hayflick and Moorhead in the 1960s (12, 13). This phenomenon, initially described as limited in the ability of human diploid cells to proliferate *in vitro*, is now recognized as a response to various stressors, including telomere shortening, oxidative stress, and chromatin structure abnormalities (14). During cellular senescence, the retinoblastoma protein (Rb) is typically dephosphorylated or hypophosphorylated via the p53/p21^WAF1/CIP1^ or p16^INK4a^/Rb signaling pathway. This process halts cell cycle progression, ultimately leading to senescence (10).

Senescent cells are characterized by the following traits (15, 16) (1): increased expression and activity of senescence-associated β-galactosidase (SA-β-gal) (2); increased levels of p21 and p16 (3); the presence of nuclear senescence-associated heterochromatin foci (SAHFs) (4); a senescence-associated secretory phenotype (SASP); and (5) an abnormally enlarged cell size and flattened morphology. Among these, the SASP is a distinctive secretory profile specific to senescent cells and is a critical marker of cellular ageing (17). Below, we extensively discuss the molecular mechanisms of ageing that may occur in different cell types.

### 2.2 Progerin accumulation and ageing

Nuclear structural abnormalities emerging during cellular senescence are dominated by progerin, a truncated form of Lamin A generated by mutations in the LMNA gene (18, 19). Progerin accumulation disrupts nuclear integrity and accelerates the aging process, especially in the cardiovascular system.

Hutchinson – Gilford progeria syndrome (HGPS) is associated with LMNA gene mutations leading to abnormal lamin levels. Patients with HGPS exhibit calcification and abrasion of vascular smooth muscle cells (VSMCs), along with significant adventitial fibrosis, leading to severe premature arteriosclerosis (20). However, progerin overexpression in different cardiac cells leads to different cardiac diseases in the HGPS mouse model. Selective overexpression of VSMC-derived progerin induces endoplasmic reticulum (ER) stress and atherogenesis (AS) (21, 22). Progerin accumulation in endothelial cells(ECs) leads to cardiac fibrosis and cardiac hypertrophy (23). Simultaneously, At the same time, progerin is also elevated in individuals with dilated cardiomyopathy, which is strongly associated with left ventricular remodeling and myocardial ageing (24).

In HGPS mouse model, the massive accumulation of prelamin A (Lamin A precursor) resulting from knockdown of Zmpste24 similarly causes nuclear lamina defects and accelerates VSMC premature senescence (25). In human arteries, prelamin A is prevalent in the media of VSMCs or atherosclerotic lesions in older individuals, whereas it rarely accumulates in young and healthy vessels. Consequently, prelamin A may emerge as a novel biomarker for cardiovascular ageing and may participate in the development of CVDs. Reducing prelamin A/progerin by injecting CRISPR/Cas9 improves HGPS symptoms in mice, which highlights a new therapeutic approach for improving age-induced CVDs (26, 27).

### 2.3 Impaired autophagy and ageing

Autophagy is able to eliminate misfolded proteins and dysfunctional organelles. Maintaining efficient autophagy is also necessary for many cellular processes associated with lifespan extension (28). Age-related decreases in autophagic activity, attributed to diminished lysosomal function as well as decreased expression of genes associated with autophagy, such as ATG7, contribute substantially to cardiovascular ageing (29–31). In VSMCs, defective autophagy accelerates aging and promotes atherosclerotic plaque formation; whereas in ECs, it exacerbates vascular inflammation and impairs NO bioavailability, thereby aggravating arterial stiffness and hypertension. In cardiomyocytes, impaired autophagy leads to accumulation of dysfunctional mitochondria and damaged proteins, which trigger myocardial fibrosis and contractile dysfunction.

Mice with impaired autophagy exhibit worsened cardiac dysfunction, whereas enhancing autophagy can enhance cardiac function and alleviate age-related heart problems by eliminating proteins with damage, dysfunctional organelles, and altered DNA (32).

Key mechanisms of autophagy include the inhibition of the target of rapamycin (mTOR) or the activation of 5-AMP-activated protein kinase (AMPK) (33). mTOR inhibits autophagy in two ways. First, mTOR directly inhibits unc-51-like kinase 1 (ULK1), which is a critical initiator of the autophagic process. Second, mTOR exerts an inhibitory effect on autophagy by hindering lysosome development, which is facilitated by impeding the nuclear translocation of TFEB (34). mTORC1, a protein complex formed by mTOR, is pivotal in the regulation of translational processes. Inhibition of mTORC1 decelerates the rate of protein translation, increasing the accuracy of mRNA translation into proteins and improving protein folding precision. This process contributes to slowing the ageing process by reducing proteotoxicity and the accumulation of oxidative stress (35, 36). The inhibition of mTOR expression to activate autophagy has been shown to suppress VSMC replicative senescence and stabilize progressive atherosclerotic plaques (37, 38).

### 2.4 Mitochondrial dysfunction and ageing

Mitochondrial dysfunction is a prominent feature of cellular senescence, primarily driven by dysregulated mitochondrial dynamics, mitochondrial DNA (mtDNA) damage, and oxidative stress (39, 40). Imbalanced mitochondrial dynamics—including hyperfusion (mediated by MFN1/2 and OPA1) and impaired fission (due to reduced DRP1/FIS1 levels)—compromise cardiomyocyte function and promote ageing (41–43). Mitochondria are the factory of cell energy, and their dynamic imbalance will damage the efficiency of ATP synthesis and produce ROS. Excessive ROS generation directly damages ECs and vascular smooth muscle cells, leading to arterial stiffening and plaque vulnerability. In cardiomyocytes, persistent mitochondrial dysfunction impairs ATP generation and activates pro-fibrotic pathways, contributing to maladaptive remodeling and heart failure. Inflammatory signaling triggered by mtDNA release through the cGAS–STING pathway further links mitochondrial senescence to chronic vascular inflammation and AS. Mitochondrial division contributes to the removal of dysfunctional mitochondria by mitophagy. Consequently, disruptions in fission-fusion balance (as evidenced by hyperfusion) accelerates the accumulation of abnormal mitochondria and oxidative proteins and triggers downstream inflammatory signaling pathways. Currently, mitochondria can promote cardiomyocyte senescence through three different mechanisms. Anderson et al. reported that excessive reactive oxygen species (ROS) production directly induces DNA and telomere damage (44, 45). Chung et al. suggested that mtDNA activates the cGAS-STING pathway, thereby stimulating SASP release (45, 46). A third view suggests that mitochondria can act on the AMPK-p53 signaling pathway, thereby accelerating cellular senescence.

### 2.5 Inflammation and ageing

The cGAS-STING pathway and SASP constitute two interrelated core elements of inflammation, a prominent feature of cardiovascular ageing. Cyclic GMP-AMP synthase (cGAS) can recognize exogenous DNA (bacterial viruses, dead cells, tumor cells, etc.) and endogenous DNA (damaged chromosomes, mitochondria, etc.) and bind to the obtained double-stranded DNA (dsDNA) to form cyclic GMP-AMP (cGAMP) (47, 48). cGAMP binds and initiates the STING protein located in the ER, initiating the activation of its downstream signaling (49, 50). CDNs, DNA damage, ER stress, and inherited gain-of-function mutations in the gene encoding STING can directly activate STING, bypassing the need for cGAMP (51, 52). Activated STING can initiate the phosphorylation and nuclear translocation of IFN regulatory factor 3(IRF3) and nuclear factor-kappa B(NF-κB), which further promotes the synthesis of IFN-I, tumor necrosis factor (TNF), and IL-6 by cells (53). These inflammatory factors are also prominent components of the SASP. Therefore, the cGAS-STING pathway contributes to inflammatory ageing by facilitating the secretion of SASP components from cells.

The mechanism of the SASP involves the activation of transcription factors such as NF-κB, C/EBPβ, and GATA4, which are closely related to the chronic DDR, and the mTOR and p38 MAPK pathways (54). Many SASP-associated genes contain binding sites for NF-kappaB and C/EBPβ b in their cis-regulatory regions, and the upregulation of expression at these sites promotes a positive feedback cycle. This cycle further consolidates the ageing state of cells by communicating with the microenvironment through NOTCH signaling, ROS, the cytoplasmic bridge, and the secretion of small extracellular vesicles (sEVs) (10).

Persistent SASP and cGAS–STING activation fuel chronic vascular inflammation, enhance endothelial dysfunction, destabilize atherosclerotic plaques, and promote myocardial fibrosis, thereby linking cellular senescence to CVDs progression.

### 2.6 Telomeres and ageing

Telomeres are protective caps at chromosome ends consisted of repetitive TTAGGG sequences and associated proteins that prevent chromosome degradation and fusion (55). These proteins help avoid the recognition of telomeres as DNA damage, initiating the DDR. Telomeres shorten as cell division repeats, and the shielding proteins no longer protect DNA after a critical telomere length is reached, thereby activating the DDR mechanism (56). This process inhibits cell cycle progression by inducing the expression of p21 and p16. The activation of the telomeric DDR (tDDR) also leads to the generation of telomere-associated DDR sites (TAFs) or telomere-induced DNA damage sites (TIFs), which are regarded as markers of tissue ageing and cellular senescence *in vitro* (57).

Activation of the tDDR and the accumulation of TAFs are also often causally linked to various age-related phenomena, including mitochondrial dysfunction, altered nutrient perception, impaired autophagy, a loss of proteostasis, and epigenetic dysregulation (57). These findings suggest that many ageing hallmarks revolve around a unified “telomere-centric” mechanistic principle (58).

### 2.7 Epigenetic regulation and ageing

Epigenetics pertains to biological mechanisms that influence gene activity without modifying DNA sequences, impacting gene expression through DNA methylation, histone modifications, and noncoding RNAs (59). DNA methylation changes gene expression through DNA methyltransferases (DNMTs) without altering the DNA sequence, affecting the cell cycle, DNA repair capacity, and cellular processes associated with cellular senescence. Thus, DNA methylation is a marker of ageing and a critical regulator of cellular ageing (60). Research indicates a link between the onset and progression of CVDs such as coronary heart disease (CHD), heart failure, and hypertension with DNA methylation (61, 62).

Modifications to histones have the ability to modify the binding strength between histones and DNA double helices while recruiting various adaptor proteins or effector proteins to remodel chromatin. Sirtuins (SIRTs) represent a group of histone deacetylases that effectively counteract ageing characteristics across various cell types (63). In cardiomyocytes, SIRT3 prevents TGFβ-induced fibrosis by activating GSK3β (64). In ECs, SIRT1 regulates endothelial nitric oxide synthase (eNOS) to mitigate oxidative damage (65) and deacetylates p65 to disrupt the interaction between acetyltransferase P300 and NF-κB, thereby inhibiting NF-kappa B activity (66).

Noncoding RNAs, including microRNAs (miRNAs) and long noncoding RNAs (lncRNAs), are vital in regulating ageing processes and CVDs. For example, when miRNA-22 is actively expressed, it accelerates the ageing and migration of cardiac fibroblasts (CFs) (67). LncRNAs bidirectionally regulate cardiac regeneration and development. Linc1405 and the lncRNAs PANCR and Hdn were found to induce the transformation of CFs into cardiomyocytes, promoting cell differentiation and heart development (68, 69). The repression of cardiac regeneration and differentiation is observed in the presence of the lncRNA CAREL (70). In summary, the regulation of cellular senescence involves various stimulatory factors and pathways (**Figure 3**).

## 3 Cardiac ageing microenvironment

Cardiovascular resident cells and immune cells together constitute the cardiac ageing microenvironment. Cardiomyocytes, ECs, VSMCs, and fibroblasts in this microenvironment undergo senescence in this microenvironment, accelerating cardiac structural abnormalities and functional deterioration. The ageing immune microenvironment includes monocytes/macrophages, dendritic cells (DCs), and T cells, which has an impact on tissue homeostasis by modulating the inflammatory response. All these cells and their interactions shape the cardiac ageing microenvironment and influence the resilience and ageing process of the heart.

### 3.1 Ageing in cardiomyocyte

**Figure 4:**
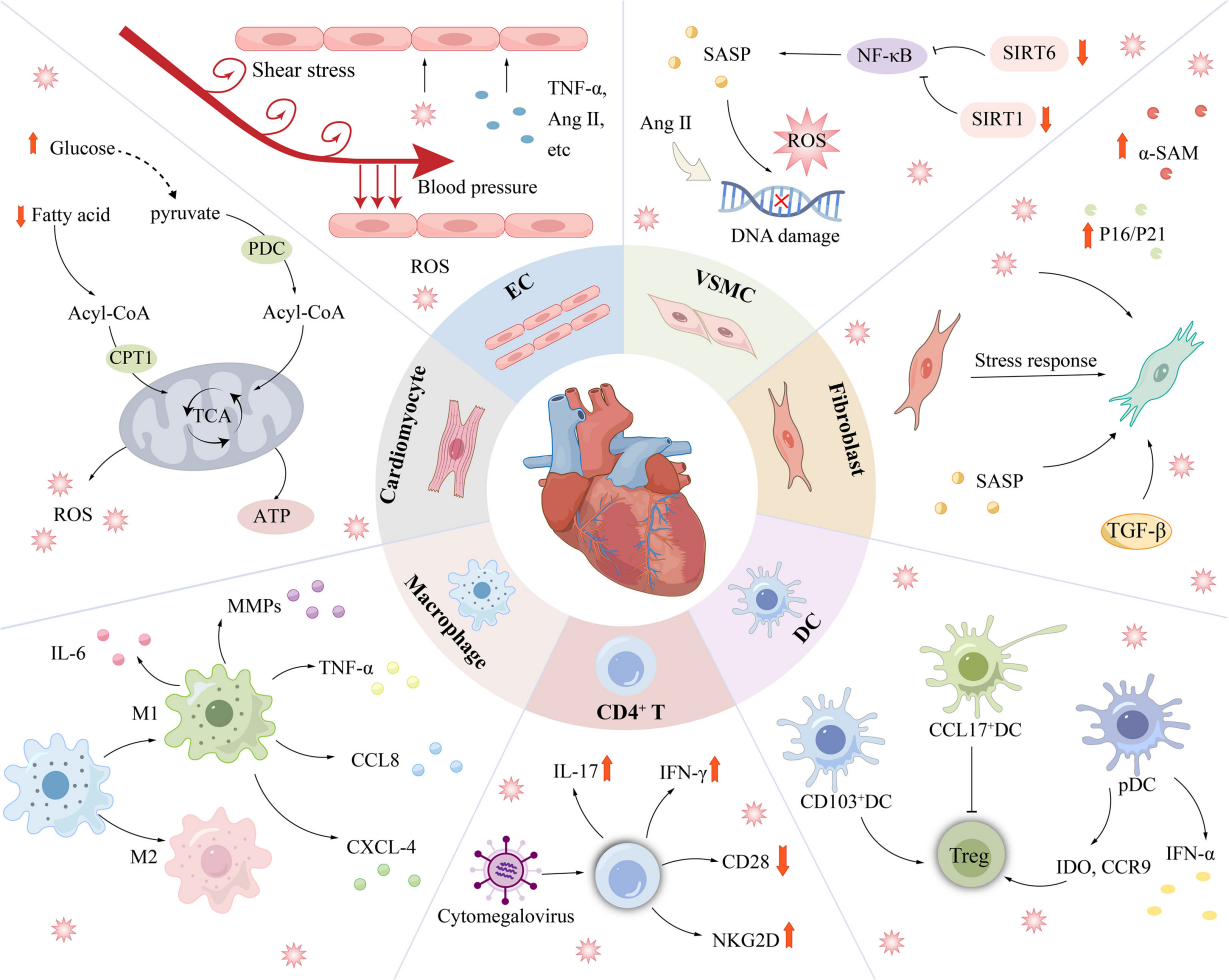
Cardiovascular disease is associated with senescence of a variety of cells. Senescent cardiomyocytes mainly showed decreased fatty acid oxidation ability and enhanced glucose oxidation pathway. ECs are continuously exposed to unique injury-stimulating environments (blood flow pressure, blood flow shear stress, circulating factors, pathogenic stimuli, etc.) and are therefore highly susceptible to injury. In addition to being affected by DNA damage, oxidative stress, etc., SIRT6 deficiency and SIRT1 inactivation can lead to senescence in VSMCs. Under stress conditions, the phenotype of CFs is irreversibly altered, as shown by an increase in ageing markers such as α-SMA. Immune-related cells such as DCs, Macrophages, and T cells regulate the progression of cardiovascular disease mainly through changes in inflammatory factors. α-SMA, myofibroblast marker; Ang II, Angiotensin II; CPT1, carnitine palmitoyl transterase-1; SIRT1/6, Sirtuin 1/6.

Multiple cells collaborate to sustain the normal physiological function of the heart. Therefore, the senescence of certain cell types increases the risk of CVDs (**Figure 4**). Cardiac cells constitute approximately 30%-40% of cardiomyocytes, which are essential for generating the force required for the heart’s pumping function (71). Cardiomyocytes that have reached senescence exhibit DNA damage, ER stress, impaired mitochondrial function, and compromised contractile performance and regulate the microenvironment through the paracrine secretion of the SASP to induce local noncardiomyocyte ageing (72). Many mechanisms that induce cardiomyocyte ageing have been identified, including telomere shortening (73), epigenetic changes (74), and the SASP (44), but metabolic dysfunction is a key factor contributing to cardiomyocyte ageing and decreased cardiac function (75).

Compared with other cells, cardiomyocytes exhibit a distinct metabolic profile, relying primarily on fatty acids and glucose for energy provision. The ratio of fatty acids and glucose in the energy supply is dynamically regulated by developmental, physiological, and pathological responses (76, 77). Fatty acyl-CoA (CoA) and pyruvate serve as the primary substrates for ATP generation in the mitochondria of cardiomyocytes and are produced through the oxidation of fatty acids and glucose, respectively. CoA and pyruvate are regulated mainly by carnitine-palmitoyltransferase-1 (CPT1) and pyruvate dehydrogenase (PDH) (78), which are rate-limiting enzymes in mitochondria. CPT1 levels are significantly reduced during ageing (79) and may lead to cardiac complications (80). A lack of CPT1 exacerbates the ageing process in cardiomyocytes and contributes to lipotoxic cardiac hypertrophy (81).

In addition, the expression levels of peroxisome proliferator-activated receptor α (PPARα) and PGC-lα, which are essential regulators of fatty acid metabolism, decrease with ageing (82). In mice prone to accelerated ageing, decreases in PPARα mRNA and protein levels lead to increases in ceramide levels, which correlate with the development of cardiac hypertrophy (83). Aged hearts demonstrate a reduced capacity for fatty acid oxidation and rely more on augmented glucose oxidation pathways to meet their metabolic needs (84).

The insulin signaling pathway is essential for glucose metabolism in cardiomyocytes and can be activated by insulin growth factor receptor (IGFR) to induce the SASP and promote cardiomyocyte senescence (85). More importantly, metabolic dysfunction impairs mitochondrial function, impacting all substrates, including increasing ROS generation (86). Defective mitochondria persist in the heart, leading to exacerbated oxidative stress and injury, alongside the activation of oxidative signaling pathways.

Senescent cardiomyocytes display diminished contractile function and disrupted conduction patterns, resulting in cardiomyopathy and arrhythmias (87). Alterations in the mitochondrial membrane potential and telomere shortening were observed in cardiomyocytes from mice with Duchenne muscular dystrophy (DMD), suggesting cellular senescence (88). Anthracyclines cause a dilated cardiomyopathy phenotype linked to cardiomyocyte senescence, as shown by increased mtDNA levels (89). In addition, ageing rat cardiomyocytes display a decreased mitochondrial membrane potential, increased ROS levels, and an attenuated ability to undergo electrical pacing, indicating an increased risk of arrhythmia (90).

### 3.2 Ageing in endothelial cell

ECs are highly active monolayers that line the inner layers of blood vessels and cover the inner surface of the entire circulatory system (71). ECs not only form the vascular barrier, which helps maintain blood flow, but also regulate vascular tone and blood pressure by synthesizing vasoactive substances and growth factors (91). However, ECs are highly susceptible to injury because they are located between circulating blood and semisolid tissues and are continuously exposed to unique injury–irritating environments (hemodynamically generated pressures, circulating factors, pathogenic stimuli, etc.). One of the consequences of EC injury is cellular senescence, which causes impaired vasodilation and vascular dysfunction. Senescent ECs can be observed in the hearts of patients with diseases such as AS, heart failure, and aneurysms (92). EC senescence is mainly caused by oxidative stress and vascular inflammation. The senescence of ECs is expedited by metabolic factors such as hyperuricemia or dysregulation of the RAAS (93). Many molecules and pathways, such as SIRT, Klotho, RAAS, IGFBP, NRF2, and mTOR, are associated with promoting EC senescence (94).

Aging and impaired function of ECs play critical roles in the development of CVDs. SIRT6 deficiency, miR-217 overexpression or NOX activity accelerate EC senescence, leading to AS (95, 96). In addition, EC senescence can mediate thrombosis by increasing plasminogen activator inhibitor-1 (PAI-1) (97). Heart failure with a preserved ejection fraction (HFpEF) represents a category of age-related CVDs closely linked to EC senescence and myocardial fibrosis (98). More importantly, mouse models of accelerated ageing have shown that EC senescence contributes to HFpEF, as evidenced by diastolic dysfunction, interstitial fibrosis, left atrial dilation, and left ventricular hypertrophy (92).

The incidence of atrial fibrillation (AF) is higher in older individuals. The onset of AF correlates with the senescence of ECs and fibroblasts (99, 100). The downregulation of eNOS and abnormalities in miRNAs are associated with EC dysfunction (101). Angiotensin II (Ang II) can induce EC senescence through an AT1R-mediated pathway, increasing ROS generation, inflammation, extracellular matrix remodeling, and vascular tone (102, 103).

### 3.3 Ageing in vascular smooth muscle cell

VSMCs are vital for regulating vascular wall tension and maintaining blood pressure (104). VSMC senescence promotes arterial stiffness and arterial calcification, leading to reduced arterial compliance and elastic reservoir dysfunction, which are the pathological foundations of diseases such as hypertension and independent risk factors for heart failure (105). Senescent VSMCs considerably influence AS development (106) and are closely associated with aortic aneurysm (107), pulmonary hypertension (108), and fibrotic neointima formation (109).

Telomere shortening, DNA damage, oxidative stress, and autophagic dysfunction can all cause VSMC senescence (110). The activation of SIRT family proteins plays a multifaceted antiaging role (111), and SIRT6 deficiency and SIRT1 inactivation can lead to senescence in VSMCs (112). Abnormal processing of Prelamin A to lamin A results in defects in the nuclear layer, increasing the vulnerability of DNA to damage and accelerating cellular senescence (25). Moreover, sustained DNA damage signals promote the transformation of VSMCs into osteoblastic vascular smooth muscle cells, leading to subsequent vascular calcification and AS (113, 114).

Interestingly, the replicative senescence of VSMCs mediates their phenotypic transformation through runt-related transcription factor-2 (RUNX-2) and induces age-related medial arterial calcification (115). In addition, senescent VSMCs exhibit elevated levels of inflammatory cytokines and reduced expression of anti-inflammatory factors (116). IL-1a activates the SASP in local cells and increases IL-6 secretion, inducing local inflammation in the cardiac microenvironment (117).

Like other heart cells, VSMC senescence also leads to CVDs, most commonly AS. Matthews et al. detected a large amount of senescent VSMCs in atherosclerotic fibrous caps (118). Compared with normal VSMCs, plaque VSMCs are distinguished by shorter telomeres, higher p16 and p21 expression, stronger SAβ-gal activity, and a flatter cell morphology. In addition, telomere shortening in intimal VSMCs is positively correlated with the severity of AS. VSMC senescence also leads to plaque instability, resulting in myocardial infarction (MI) and stroke. This instability may be related to the secretion of MCP1, MIP1a/b, and CCL3/4, which promote the accumulation of monocytes, macrophages, and lymphocytes (119, 120). Ang II also induces premature VSMC senescence, thereby accelerating the development of AS (103). The overexpression of TRF2 decreases DNA damage and inhibits senescence in VSMCs, thereby attenuating plaque vulnerability (119).

Additionally, VSMC senescence may also participate in the pathophysiological processes of pulmonary arterial hypertension through the SASP (121). The existing literature suggests that VSMC senescence is associated with the development of aortic aneurysms. Liao et al. were the first researchers to document that medial VSMCs from patients with AAA display enhanced replicative senescence. Compared with VSMCs from the same patient’s inferior mesenteric artery (IMA), AAA-derived VSMCs are more extensive and rounder, and their proliferative capacity is significantly diminished (122). Angiotensin converting enzyme, Ang II, and RAS accelerate VSMC ageing and lead to the formation of AAAs by stimulating the production of proinflammatory cytokines, ROS, and the ageing phenotype in VSMCs (123).

### 3.4 Ageing in cardiac fibroblasts

CFs are important components of cardiac noncardiomyocytes. CFs maintain the extracellular matrix (ECM) structure and adhesion integrity by expressing integrins and matrix metalloproteinases (MMPs) (124). In addition, CFs can also participate in paracrine secretion to regulate the hypertrophy, proliferation, growth, and ageing of surrounding cells (125).

Under stress conditions, CFs change their phenotype and transform into myofibroblasts. CFs undergo irreversible senescence upon sustained stimulation by stressors. Notably, the expression of ageing biomarkers such as p16 and p21 is significantly increased in the hearts of mice following MI (126). Costaining of α-SMA (a marker for myofibroblasts) with p53 or p16 revealed an increased presence of senescent fibroblasts within the border zone of the infarct (127). Similarly, senescent fibroblasts have been detected in mouse models of cardiac hypertrophy and remodeling (128). In conclusion, senescent fibroblasts are ubiquitous in fibrotic areas and are involved in the pathological processes associated with myocardial fibrosis.

CF senescence has a dual impact on cardiac health. On the one hand, as cardiac fibroblasts enter a senescent state, their ability to secrete collagen decreases, which may delay the initial stage of the wound healing process. On the other hand, fibrosis can be reduced and cardiac function can be improved by inducing CF senescence. Conversely, if the natural ageing process of fibroblasts is delayed, it may exacerbate the degree of myocardial fibrosis and ultimately lead to cardiac dysfunction. Therefore, balancing the ageing of fibroblasts is essential for maintaining heart health.

Following acute MI, the activated cardiac fibroblast phenotype undergoes dynamic changes from an inflammatory to a noninflammatory state, driving extracellular matrix regulation and ultimately supporting scar formation (129). Premature ageing of CFs reduces the production of ECM components, such as collagen, and may lead to the inhibition of reparative fibrosis in wounds during healing. However, under chronic pressure loading, the premature ageing of CFs may play a protective role by reducing ECM deposition and preventing excessive fibrosis, thereby preventing further decreases in cardiac stiffness and function (127, 128). Furthermore, the overexpression of matricellular protein (CCN1) may induce CF senescence, thereby reducing myocardial fibrosis and enhancing cardiac function post-MI, thus playing a beneficial role in acute ischemia (84). These findings suggest potential positive effects of fibroblast senescence in some cases.

However, some studies indicate that the beneficial effects of CF senescence require a balance with the potentially deleterious effects of ageing. Gavin D. Richardson et al. found that following myocardial ischemia/reperfusion injury (IRI), biological processes associated with fibrosis and inflammation are attenuated upon the administration of the antiaging agent navitoclax, thereby improving cardiac function and reducing the scar size (130).

Interestingly, NEIL3 is an enzyme involved in DNA repair processes that minimizes oxidative damage to DNA by recognizing and removing oxidized bases. CFs proliferate excessively in the hearts of Neil3 ^−/−^ mice, but the risk of cardiac rupture remains (131). DNA damage caused by Neil3 deletion may initiate the ageing phenotype in the cardiac microenvironment via SASP-mediated paracrine signaling. This process increases MMP2 expression, leading to ECM degradation and, ultimately, cardiac rupture (131, 132). Cardiac fibrosis tends to increase with age and is correlated with HFpEF (133). Some molecules, such as miR-1468-3p and SIRT6, promote the ageing of CFs by regulating TGF-β1 signaling, which in turn increases the occurrence of myocardial fibrosis (134). These studies suggest that regulating the ageing balance of fibroblasts is crucial for treating CVD.

### 3.5 Ageing in monocyte/macrophage

Stoneman et al. showed that the quantity of monocytes/macrophages significantly promote the development of atherosclerotic plaques, including increasing the collagen content in plaques and the formation of necrotic cores (135). Monocytes undergo a metabolic shift toward glycolysis and enhance pro-inflammatory signaling upon stimulation with oxidized low-density lipoprotein (ox-LDL) (136). Within the intima, these monocytes differentiate into macrophages under macrophage colony-stimulating factor (M-CSF) regulation (137). The resulting M1 macrophages promote inflammatory responses through abundant secretion of growth factors and cytokines, particularly TNF-α and IL-1β - two central mediators of atherosclerosis-related inflammatory pathways (138). These activated M1 macrophages further stimulate CFs via the Smad3 signaling pathway by releasing profibrotic factors (particularly TGF-β1), thereby upregulating collagen and MMPs production, which ultimately leads to abnormal extracellular matrix deposition and remodeling (139). In contrast, M2 macrophages exhibit anti-inflammatory properties through IL-4, IL-13, and IL-10 secretion.

Ageing macrophages have a greater effect on plaque formation. Senescent macrophages can undergo polarization towards the M1 phenotype and release SASP factors, including TNF-α, IL-6, IL-1β, CCL2, and MMP 9, the collagenase enzyme. In addition, senescent macrophages have impaired efferocytosis capacity, increasing the expansion and vulnerable plaque shape of necrotic cores (140). Their collective actions contribute to the accelerated advancement of atherosclerotic plaques (141). Senescent macrophages accumulate in the subendothelial area during the early stage of AS and drive the pathological development of AS by increasing the expression of inflammatory cytokines and chemokines. In the late stages of AS, macrophages increase plaque instability, which is characteristic of elastic fiber fragmentation and fibrous cap thinning, by increasing metalloproteinases (142).

### 3.6 Ageing in Dendritic cell

In CVDs, DCs act as antigen-presenting cells (APCs) to influence the progression of AS by regulating Tregs (143). Senescent DCs exhibit downregulated expression of MHC-I/II molecules, leading to impaired T cell activation and compromised immune surveillance functions. However, different DC subsets present within the vessel wall each have unique functions, which reflect their diversity and complexity in CVDs. For example, CD103^+^ DCs are present in the normal arterial wall and exert anti-AS effects mainly by inducing Tregs, whereas CCL17^+^ DCs exert pro-AS effects mainly by limiting Treg production. In Ldlr^−/−^ mice, impaired autophagy in CD11b^+^ DCs due to Atg16l1 deficiency promotes aortic CD4^+^ Treg cells expansion and reduced AS (144). The role of pDCs in regulating AS is also complex. On the one hand, pDCs promote Treg differentiation by releasing indoleamine 2,3-dioxygenase (IDO) and chemokine (C-C motif) receptor 9 (CCR9), thereby producing IL-10 and mitigating AS progression (145). On the other hand, pDCs also accelerate AS formation by producing IFN-α.

Notably, senescence is associated with increased DC activation and lipid contents in DCs compared with the characteristics of DCs in young adult and aged mice. The regulation of lipid accumulation and activation of DC subsets may be attributed to the decrease in the response to infection with ageing (146). Although increased accumulation of DCs and Tregs has been reported in the murine atherosclerotic intima, the role of senescent DCs in CVD development remains unclear (147).

### 3.7 Ageing in T cell

During the development of AS, antigen-presenting cells (APCs) present antigens produced from components such as LDL to naïve CD4^+^T cells. This process results in the stimulation of antigen-specific CD4^+^T cells and the secretion of the proinflammatory cytokines IFN-γ and TNF or the anti-inflammatory cytokine IL-10 to regulate macrophage polarization (148). Therefore, T cells are bifaceted in the regulation of the establishment and stability of atherosclerotic plaques, which can not only exert beneficial inhibitory effects but also contribute to facilitating the formation of plaques.

Ageing T cells are associated with CVD pathological progression. In older individuals, an increase in CD4+ T-cell populations with high expression levels of IL-17 and IFN-γ has been observed. These cells also display characteristics commonly associated with ageing, such as decreased CD28 expression and elevated NKG2D levels. Interestingly, these changes are strongly linked to metabolic risk factors for CVDs (149). Recent findings have shown that cytomegalovirus (CMV) seropositivity, a widely recognized driver of T-cell senescence, is closely linked to the incidence of CHD. Additionally, there is a positive correlation between CMV seropositivity and the risk of stroke, MI, and mortality from CVDs (150, 151).

Furthermore, the presence of aged T cells in the bloodstream is linked to disease relapse and the emergence of additional CVDs among individuals diagnosed with acute coronary syndrome (152). Indeed, the detrimental impact of ageing-related T cells on CVDs has been documented in mice. Specifically, in a mouse model of hypertension induced by Ang II, the introduction of T cells from aged mice into young recipients expedited cardiac and renal damage through an increase in IFN-γ secretion, thereby fostering inflammation and fibrosis. A recent study revealed that ageing-related cardiovascular changes, such as aortic dilatation, partial rupture, and myocardial dysfunction, developed in a mouse model of premature T-cell failure due to mitochondrial dysfunction (153). The results of this study suggest that the presence of aged T cells may directly impact the progression of CVDs.

## 4 Endocrine ageing and sex-specific differences in cardiovascular ageing

Endocrine ageing, a core aspect of the biology of aging, has garnered increasing attention. Research indicates that the decline in estrogen, testosterone, growth hormone (GH), and thyroid hormone (TH) levels is closely associated with cardiovascular dysfunction, increased vascular stiffness, elevated inflammation, and myocardial remodeling. Furthermore, sex differences are evident throughout the spectrum of cardiovascular disease. Women experience relatively stronger cardiovascular protection before menopause, but this risk rises rapidly post-menopause. In contrast, men exhibit a higher vascular risk profile due to age-related declines in androgen from midlife onward. These findings suggest a significant interaction between endocrine aging and biological sex differences in the process of cardiovascular ageing.

### 4.1 Estrogen and cardiovascular protection

Sufficient literature demonstrates that estrogen exerts multiple protective effects on the cardiovascular system, including promoting vasodilation, protecting endothelial function, improving lipid metabolism, reducing inflammation and mitigating oxidative stress (154). Estrogen primarily exerts its pleiotropic protective effects through nuclear receptors (ERα/ERβ) and membrane-associated receptors (GPER).

In the regulation of vascular tone, ERα rapidly activates eNOS through the PI3K/Akt signaling pathway, mediating the rapid release of NO from ECs (155).NO serves as a crucial vasodilator that effectively dilates blood vessels, improves endothelial function, and exerts anti-atherosclerotic effects. Conversely, reduced NO levels diminish vascular antioxidant capacity and exacerbate inflammatory responses.

Estrogen exerts a positive regulatory effect on lipid metabolism. It enhances the production of high-density lipoprotein (HDL) by inhibiting hepatic lipase activity and accelerates the clearance of low-density lipoprotein (LDL) through upregulation of LDL receptor expression. During the menopausal transition, decreased estrogen levels accompanied by a relative increase in androgen levels may lead to disordered lipid metabolism, thereby increasing the risk of AS (156). Estrogen regulates lipid metabolism mainly through genomic and non-genomic effects mediated by estrogen receptors (ERs). Among them, ERα mainly promotes the transport of cholesterol from peripheral tissues (such as arterial wall macrophages) to the liver by regulating apolipoprotein E (APOE) and cholesterol reverse transporter ABCA1/ABCG1, thereby enhancing HDL biosynthesis (157).

In addition, estrogen plays a role in vascular protection through various mechanisms such as anti-oxidation, promoting NO production and inhibiting inflammatory signaling pathways. Activation of ERα can inhibit NF-κB and NLRP3 inflammasome signaling pathways, reduce the release of inflammatory factors such as IL-6 and TNF-α, thereby reducing vascular endothelial inflammation (158). ERβ inhibits LDL oxidative modification by enhancing the activity of superoxide dismutase (SOD) and glutathione peroxidase (GPx), reducing the accumulation of reactive ROS (159).

Although a large amount of evidence supports that estrogen has a protective effect on the cardiovascular system, the clinical application of its alternative therapy (ERT) is still controversial. A number of large-scale clinical trials have suggested that ERT may increase the risk of stroke and thromboembolic events, so its benefits and safety should be carefully evaluated in translational applications (160).

### 4.2 Androgens and cardiovascular homeostasis

The effect of androgen on CVDs is a complex and controversial topic. However, most studies suggest that elevated TES levels have a protective effect on the cardiovascular system.

Early clinical studies have found that the incidence of hypertension and coronary artery disease in men is higher than that in premenopausal women, thus forming the view that TES and other androgens may be detrimental to cardiovascular health (161). However, the latest clinical and animal research evidence overturns the traditional understanding that androgens have significant benefits for male blood pressure and metabolism - both of which are key risk factors for CVDs (162). Systematic follow-up evaluations of early epidemiological investigations, clinical studies, and animal experiments revealed that these initial studies had many methodological flaws in experimental design, model selection, and data analysis (163, 164). Epidemiological studies have shown that low androgen levels are an independent risk factor for CVDs (165, 166). Low TES is often accompanied by lipid metabolism disorders, insulin resistance and central obesity.

The protective effect of TES on the heart is mainly manifested in its diastolic vascular function and endothelial protection. The core mechanism of TES relaxing blood vessels is to activate cGMP-PKG signaling pathway by promoting NO synthesis, and then open BKca channel (167, 168).In rat aortic tissue, TES significantly enhances NO synthesis through the androgen receptor and calcium influx, whereas the calcium channel blocker verapamil attenuates TES-induced NO production (169). Cardiovascular ageing is closely associated with reduced NO synthesis in ECs. Androgens help counteract this process by promoting eNOS activity and NO production, thereby enhancing the antioxidant capacity of ECs—a mechanism aligned with cardiovascular anti-ageing pathways.

At physiological levels, androgens can improve endothelial function and enhance antioxidant capacity. However, supraphysiological doses may lead to adverse effects, such as hypertensive heart disease, increased risk of venous thrombosis, and recurrence in patients with prostate cancer (170, 171). However, meta-analyses have also indicated that TES replacement therapy is safe in the short to medium term, with no higher risk of cardiovascular events compared to men not receiving TES treatment (172). In summary, current clinical evidence is insufficient to support the beneficial effect of androgen replacement therapy on CVDs, and further large-scale clinical trials are needed to evaluate its efficacy and safety.

### 4.3 Growth hormone and the cardiovascular system

With the increase of age, the secretion of growth hormone (GH) decreases gradually. Some elderly people have age-related GH deficiency. Since the GH/IGF-1 axis plays a critical role in the development and functional regulation of the cardiovascular system, reduced GH secretion is considered to be closely associated with metabolic disorders and an increased risk of CVDs.

The GH/IGF-1 axis maintains cardiac structure and metabolic homeostasis by promoting myocardial gene expression, enhancing amino acid uptake and protein synthesis, and regulating cardiomyocyte size. It upregulates muscle protein mRNA, augments type I calcium channel activity, improves calcium sensitivity, and increases Ca ² -ATPase levels, thereby optimizing calcium handling and contractility (173). Physiological GH/IGF-1 signaling is crucial for normal heart mass and function.

In the vascular system, GH/IGF-1 receptors are widely expressed. Experimental studies indicate that GH/IGF-1 exerts angiogenic factor-like effects by inducing the proliferation and migration of vascular endothelial cells and promoting the formation of new capillaries (174). Furthermore, it enhances vascular endothelial function and regulates vasomotion through stimulating NO synthesis, thereby playing a key role in maintaining vascular homeostasis.

In addition, GH exerts metabolic effects including promoting protein synthesis, stimulating lipolysis, and suppressing glucose utilization. It also modulates vascular tone, thereby influencing peripheral resistance and blood pressure. Consequently, abnormal GH secretion not only contributes to metabolic disorders but may also disrupt blood pressure homeostasis, elevating the risk of atherosclerosis and other cardiovascular diseases.

Clinical evidence indicates that GH replacement therapy improves the lipid profile (reducing LDL-C and increasing HDL-C), restores vascular endothelial function, and lowers inflammatory markers—such as high-sensitivity C-reactive protein, IL-6, and TNF-α—in patients with growth hormone deficiency, while also reducing carotid intima-media thickness (175). Some studies further suggest that GH treatment can enhance cardiac function, exemplified by reduced left ventricular end-systolic volume and improved ejection fraction (176). However, large-scale prospective clinical trials using cardiovascular events as primary endpoints are still lacking, and the long-term cardiovascular benefits of such therapy require further validation (177).

### 4.4 Thyroid hormone and cardiovascular system

Thyroid hormones (TH) play a critical role in maintaining cardiovascular homeostasis by regulating heart rate, myocardial contractility, and systemic vascular resistance. Thyroid dysfunction is frequently observed in patients with CVDs, with subclinical hypothyroidism (SCH) being the most common form (178). Epidemiological evidence consistently indicates that the prevalence of ​​overt hypothyroidism​ and SCH increases with advancing age and is strongly associated with dyslipidemia, hypertension, diabetes, and other cardiovascular risk factors (179–181).

Thyroid dysfunction affects cardiovascular function by altering the levels of T3, T4 and TSH. T3 binds to nuclear thyroid hormone receptors (TRs) in cardiomyocytes, promoting the synthesis of contractile proteins such as myosin heavy chain V3, and enhances myocardial contractility by upregulating β1-adrenergic receptor expression (182). In addition, T3 increases intracellular cAMP levels, which upregulates Ca^2+^-ATPase activity and thereby improves diastolic relaxation. Moreover, thyroid hormones can activate the PI3K/AKT signaling pathway to stimulate NO production in vascular endothelial cells, ultimately reducing systemic vascular resistance (183).

Thyroid hormone also regulates lipid metabolism by acting on genes such as the LDL receptor (184). TSH is positively associated with elevated lipids, insulin resistance, and hyperglycemia. TSH not only affects lipid metabolism indirectly by regulating TH levels, but also acts directly on hepatic TSH receptors to activate cAMP/PKA/CREB signaling pathways and promote cholesterol synthesis. This explains the phenomenon that SCH patients have elevated lipids despite normal TH levels (185).

Levothyroxine is a commonly used drug for the treatment of hypothyroidism. Available studies have shown that levothyroxine replacement appears to improve left ventricular function, endothelial function, and lipid metabolism and partially reverse the pathological effects of hypothyroidism on the cardiovascular system (186). However, there remains a lack of consistent evidence for its cardiovascular benefit in SCH patients, which needs to be verified by further large-scale prospective studies.

In general, these changes in hormone levels directly or indirectly contribute to the development and progression of CVDs mainly through the regulation of lipid metabolism, the impact of inflammatory factors, and the SASP. From the therapeutic perspective, although hormone replacement therapy (HRT) and selective estrogen receptor modulators (SERMs) can improve endothelial function and lipid metabolism disorders, their long-term safety remains controversial. Although TES replacement therapy is increasingly active, the results of studies on cardiovascular outcomes are variable and require strict weighing of risks versus benefits. Compared with systemic sex hormone intervention, targeting the clearance of senescent cells and SASP may more accurately and safely intervene in the endocrine ageing process.

## 5 Targeting aging to alleviate CVDs

A large body of data suggests that ageing cardiovascular cells add to and accelerate the development and progression of CVDs. Hence, the targeted clearance of senescent cells represents a promising therapy for averting or managing age-related ailments such as CVDs (187). While senescent cells can originate from various tissues, diseases, and cell types, they exhibit common ageing mechanisms and biochemical characteristics, which opens the possibility of treating or delaying ageing-related diseases by removing senescent cells. As early as 2004, a report noted that the burden of senescent cells in mammals is inversely proportional to their healthy lifespan. This insight has prompted researchers to explore the development of targeted therapies to eradicate these ageing cells (188). Since then, the therapeutic elimination of senescent cells has emerged as a groundbreaking strategy to decelerate ageing and potentially inhibit disease progression.

### 5.1 Drug therapy

#### 5.1.1 Senolytics: selective removal of senescent cells

Senolytics, compounds designed to target and eliminate senescent cells selectively, facilitate this process primarily by inhibiting antiapoptotic factors. In 2015, the Kirkland trial at the Mayo Clinic in the United States first reported the first group of senolytics, dasatinib and quercetin (**Table 1**) (206). Dasatinib, a commonly employed medication for leukemia treatment in clinical settings, effectively inhibits both Bcr-Abl fusion gene I and Src tyrosine kinase (207). Quercetin, a flavonol compound, can suppress PI3K activity, increase SIRT1–213 expression (208) and impede mTOR signaling (209). The combination of dasatinib and quercetin (D+Q) enhances the clearance of senescent cells and promotes improvements in cardiac function and carotid vascular reactivity in older mice.

**Table 1:** Senolytics treatment in age-related diseases.

Compounds	Target (or targets)	Age-related diseases	Developtment status	Refs
Dasatinib (D)and Quercetin (Q)	Pan-receptor tyrosine kinases	Alzheimer disease	Clinical trial: NCT04685590	(189)
		Idiopathic pulmonary fibrosis	Clinical trial: NCT02874989	(190)
		Chronic kidney disease	Clinical trial: NCT02848131	(191)
Navitoclax (ABT-263)	Bcl-2, Bcl-X_L_ and BCL-W	Ovarian cancer	Clinical trial:NCT02591095	(192)
		RAS-mutant tumors	Clinical trial: NCT02079740	(193)
ABT737	BCL-X and BCL-W_L_	Aged lungs and skin	in vivo experiment: K5-rtTA/tet-p14 transgenic mice	(194)
A1331852 and A1155463	BCL-X_L_	NA	in vitro experiment: HUVECs and IMR90 cells	(195)
Piperlongumine(PL)	Unknown	NA	in vitro experiment: human WI‐38 fibroblasts and senescent cells	(196)
17-DMAG	HSP90	Human progeroid syndrome	in vivo experiment: Ercc1^-/Δ^ mice	(197)
		Nephropathy and Atherosclerosis	in vivo experiment: apoE^-/-^ mice	(198)
Fisetin	PI3K-mTOR	Progeria	in vivo experiment: f1 p16^+/Luc^; Ercc1^−/Δ^ mice	(199)
Curcumin	NF-κB	Neurodegenerative disease	Clinical trial: NCT01383161	(200)
Cardiac glycosides (ouabain, digoxin)	Na+ /K+ -ATPase	Lung fibrosis	in vivo experiment: immunodeficientnude NMRInu/nu mice	(201)
		Tumor cells and senescent cells	in vivo experiment: C57BL/6J mice	(202)
	retinoid-related orphan receptor-γ	Atherosclerosis	in vivo experiment: ApoE^-/-^ mice	(203)
FOXO4-DRI	FoxO4-p53		in vivo experiment: Xpd^TTD/TTD^ mice	(204)
procyanidin C1 (PCCI)	NOXA and PUMA(BCL-2 Member)	NA	in vivo experiment: C57BL/6L mice	(205)

Interestingly, at the time, the Kirkland team noticed an essential phenomenon: the activity of proapoptotic pathways increased significantly in senescent cells. Based on this result, they proposed a bold hypothesis: senescent cells rely on senescent cell antiapoptotic pathways (SCAPs) to antagonize apoptosis, thus allowing them to eventually survive (206). The theoretical hypothesis of SCAPs proposed by the Kirkland team at the time was confirmed by a series of subsequent studies in multiple laboratories; at the same time, many novel senolytics emerged based on this feature of senescent cells (**Table 1**).

Subsequently, navitoclax (ABT-263), an inhibitor of the synthetic BCL-2 protein family (which includes Bcl-2, Bcl-XL, and Bcl-w), was identified as a third-generation senolytic drug (210, 211). Experiments performed by Childs et al. demonstrated that the depletion of senescent cells by ABT-263 (navitoclax) significantly inhibited AS in the aortic arch of Ldlr^−/−^mice (142). ABT-263 also promotes the clearance of senescent cardiomyocytes, thereby reducing myocardial fibrosis and cardiomyocyte hypertrophy (212). ABT-263 administration in mice with simulated MI alleviates myocardial remodeling, enhances diastolic function, and increases the overall survival of aged mice (213).

Piperlongumine (PL) is also a senolytic that promotes apoptosis in senescent cells. PL kills WI-38 fibroblasts, but does not induce ROS generation, by inducing apoptosis (196). The combined use of PL with ABT-263 resulted in enhanced antiaging activity. These findings suggest that we can reduce the dose of ABT-263 when administered in combination with the other two drugs, significantly reducing the adverse effects of ABT-263. However, the antiaging mechanism of PL needs to be clarified. Notably, senescent cells share survival traits with cancer cells. Thus, PL has shown promise in inducing apoptosis in these cells by suppressing the PI3K/Akt/mTOR signaling pathway (206).

In 2017, scientists such as Kirkland discovered that drugs such as fisetin and the BCL-XL inhibitors A1331852 and A1155463 also have basic antiaging effects (195). In recent years, a growing array of senolytics with antiaging potential has been identified, including sexual small molecules, natural products and their key components, as well as peptide inhibitors designed to target known SCAPs (e.g., FOXO4-DRI) (204). FOXO4-DRI can interfere with the interplay between FoxO4 and p53 in senescent cells and trigger apoptosis in senescent but unhealthy cells by releasing and activating p53.

Interestingly, most reported senolytics appear to clear only one or several specific types of senescent cells. For example, fisetin explicitly triggers programmed cell death in aged human umbilical vein endothelial cells (HUVECs). However, it does not have any senescence-inducing effects on aged IMR90 cells, human lung fibroblast lines, or primary human preadipocytes (195). Navitoclax, A1331852, and A1155463 exhibit the ability to trigger programmed cell death in aged HUVECs and IMR90 cells but show limited efficacy in inducing apoptosis in senescent preadipocytes (214). In contrast, dasatinib selectively induces apoptosis in senescent human preadipocytes more efficiently than in HUVECs (206). Individual senolytic drugs have different effects even when they act on a specific type of cell. For example, navitoclax has apoptosis-inducing effects on senescent embryonic fibroblasts such as IMR-90 cells. However, its efficacy is relatively low for senescent primary lung fibroblasts (211). Hence, accurately defining or drawing conclusions about the generalizability and effectiveness of particular senolytics without thorough empirical examinations is difficult.

A recent study revealed that procyanidin C1 (PCC1) can safely and efficiently clear various cell types and senescent cells generated by different senescence triggers (205). In addition, PCC1 significantly improved the physiological function and lifespan of ageing mice, and the creatinine, body weight, urea and immunity of the mice were not affected throughout the process. Phytochemical senolytics of natural origin, similar to PCC1, deserve in-depth exploration as potential antiaging agents.

#### 5.1.2 Senomorphics: SASP inhibition

The SASP contributes to both the generation of senescent cells and the enhancement of senescence within the microenvironment through paracrine and autocrine signaling mechanisms. Senomorphics, which inhibit the SASP without killing senescent cells, are another approach to alleviate tissue disturbances, organ regression, and body ageing caused by cellular ageing.

Senomorphics can lower SASP expression levels in senescent cells either directly or indirectly. This process is achieved by inhibiting various transcription factors, such as NF-κB, the JAK2/STAT3 signal transduction pathway, the TRAF6/TAK1 inflammatory signal transduction pathway, the mTOR protein kinase, and other signaling pathways involved in inducing and sustaining the SASP (215).

Prior research has demonstrated that NF-κB inhibitors can reduce the expression of proinflammatory components of the SASP, especially cytokines and chemokines (216). Resveratrol and epigallocatechin gallate (EGCG) are both NF-κB inhibitors. The former downregulates the levels of SASP-related proinflammatory cytokines such as IL-8 and TNF-α by inhibiting the SIRT1/NF-κB signaling pathway (217). The latter can directly downregulate the production of TNF-α and IL-6 in 3T3-L1 preadipocytes (218).

Similar natural compounds include naringenin, apigenin, pterostilbene, kaempferol, and catechin, which are relatively safer than synthetic compounds and have better application prospects (219, 220). Rapamycin and its analogues (rapalogs), on the other hand, reduce SASP expression levels by inhibiting mTOR activity and can prolong the healthy lifespan and overall lifespan of mice (221, 222). Metformin, a drug that effectively treats the symptoms of individuals with type 2 diabetes mellitus (T2DM), can inhibit SASP expression and alleviate age-related chronic diseases (223). It impedes tumor development by reducing SASP production through the inhibition of IKK/NF-κB activity (224). Ruxolitinib, a tyrosine kinase inhibitor, is a JAK1/JAK2-STAT3 pathway-targeting agent that inhibits the development and progression of the SASP *in vitro* and *in vivo* (225). In a population of older individuals diagnosed with myelodysplastic syndrome and a median age of 65 years, the administration of ruxolitinib alleviated the intensity of asthenia symptoms, encompassing factors such as weight, strength, and excessive appetite (226).

However, the issue that arises from the inhibition of intracellular pro-SASP signaling is the potential increase in cancer risk due to the disruption of SASP factor expression. For example, in mouse lymphoma models, downregulation of the SASP by the inhibition of NF-κB attenuates immune surveillance following therapeutic ageing and synergizes with p53 insufficiency to lead to ageing escape, resulting in treatment resistance and relapse (216). Future in-depth clinical studies specifically addressing these issues are still needed.

Although so many senolytics and senomorphics have been found, most of them are still in the stage of *in vitro* and animal experiments (**Table 1**). Animal models offer the advantage of rapidly validating theories and mechanisms, but their limitation lies in the gap from the pathological ageing environment in humans. More importantly, in the same aging research, different laboratories may use natural aging mice, transgenic models, drug-induced models, etc., resulting in significant differences in the effect of drugs on scavenging senescent cells. Moreover, the disease course in animal models progresses much faster than human natural ageing, which may exaggerate drug efficacy or obscure long-term adverse effects. In addition, many senolytics or senomorphics demonstrate promising results in animal studies, but their effectiveness is highly dependent on cell type. For instance, navitoclax effectively eliminates senescent VSMCs in mice, yet shows limited efficacy in other cell types.

At present, clinical trials of anti-ageing drugs targeting CVDs have not yet been initiated and remain largely confined to metabolic diseases or osteoarthritis. Although theoretical foundations and animal experiments provide important mechanistic insights, the results should be interpreted with caution when translated into clinical practice. The development of anti-aging drugs specifically targeting CVDs will still require a long process of exploration.

### 5.2 Increasing immune surveillance of senescent cells

Under physiological conditions, senescent cell clearance primarily relies on apoptosis and immune-mediated mechanisms. However, most senescent cells acquire anti-apoptotic properties, rendering the immune system crucial for their elimination. Currently, two principal immunotherapeutic strategies exist for cardiac injury repair: molecular-level interventions targeting IL-1β to mitigate inflammatory responses, and cellular-level approaches utilizing senescence-specific ligands to direct immune cell-mediated recognition and clearance.

The IL-1β-IL6-CRP axis constitutes a central inflammatory pathway in atherosclerosis and cardiovascular disease, where IL-1β serves as the upstream regulator that activates the NLRP3 inflammasome to induce ECs expression of adhesion molecules, thereby promoting inflammatory cell recruitment and macrophage activation. Furthermore, IL-1β enhances IL-6 production, which stimulates hepatic synthesis of CRP, fibrinogen, and plasminogen activator inhibitors (227). The CANTOS clinical trial demonstrated that IL-1β inhibition with canakinumab significantly reduced cardiovascular risk in patients with elevated inflammation (hs-CRP >2 mg/dL) independent of LDL modulation, but there were limitations of increased risk of infection and no improvement in mortality (228). Conversely, the broad-spectrum anti-inflammatory drug methotrexate showed no cardiovascular benefit, confirming the need for pathway-specific interventions (229). These findings highlight the therapeutic potential of developing novel interventions against specific pathogenic pathways in AS, including chemokine-receptor interactions, immune checkpoint, immunemetabolic modulation, and hormonal/lipid mediator networks, which may collectively overcome the limitations of conventional anti-inflammatory strategies while providing more precise therapeutic effects (230).

Emerging findings indicate that various immune cells, such as macrophages, NK cells, neutrophils, and cytotoxic T cells, are involved in the immunosurveillance of senescent cells (231). Senescent cells recruit corresponding immune cells for recognition and clearance by expressing different ligands on their surface. For example, senescent IMR-90 fibroblasts increase the expression of MICA and ULBP2, the corresponding ligands of the NK cell-activating receptor NKG2D, which triggers their targeted clearance by NK cells (232). Furthermore, specific markers such as major histocompatibility complex class II (MHCII) molecules may be expressed by senescent cells, enabling their precise identification and subsequent elimination by CD4^+^ T cells within the immune system (233). However, how these immune cells clear apoptotic or senescent cardiovascular cells remains unknown. Notably, chimeric antigen receptor (CAR)-T cells may be a potential immune surveillance tool for ageing.

The efficacy and specificity of cytotoxic T cells decline with age, despite their crucial roles in identifying and eradicating foreign entities within the human body. CAR-T cells represent a form of live-cell therapy that allows T cells to more precisely identify cancer cell surface markers by introducing chimeric antigen receptors (CARs) onto the surface of T cells using genetic engineering techniques. This technology has already shown considerable efficacy in the treatment of a range of cancers (234) and is FDAI1-approved for treating certain leukemias and lymphomas (235). In recent years, CAR-T-cell therapy has been considered to target the elimination of noncancerous cells, such as senescent cells.

Fortunately, CAR-T cells have progressed successively as antiaging drugs. High expression of fibroblast-activating protein (FAP) in CFs leads to myocardial fibrosis and myocardial disease. A reversal of cardiac fibrosis and restoration of function were observed in mice exposed to Ang II and phenylephrine following the adoptive transfer of FAP-targeting CD8+ T cells generated using CAR-T-cell technology (236). Corina Amor et al. reported that urokinase-type plasminogen activator receptor (uPAR), a cell membrane protein, is significantly upregulated with age. They have also successfully documented the efficacy of CAR-T cells targeting uPAR in clearing senescent cells both *in vivo* and *in vitro* (237). UPAR-CAR-T cells improve exercise capacity, reverse liver fibrosis, and ameliorate metabolic dysfunction in aged mice and mice fed a high-fat diet (237).

Unlike senolytics, which are not system-specific and require long-term repeated administration, uPAR-CAR-T cells demonstrate enhanced targeted clearance and can achieve long-term therapeutic and preventive effects with a single low dose administration (238). These findings confirm the strong therapeutic activity of antiaging CAR-T cells in addressing ageing-related disorders. Notably, XuDong Zhao et al. recently reported that NKG2D ligand (NKG2DL) was upregulated in senescent cells (239). Accordingly, the team developed NKG2D-CAR-T-cell therapy that selectively targets the consumption of NKG2DL-expressing senescent cells in mice and juvenile nonhuman animals while improving the function of multiple organs. Nevertheless, pertinent evidence indicating that ageing cardiovascular cells can produce NKG2DL is insufficient. In summary, the utilization of specialized CAR-T cells for the targeted elimination of aged cardiovascular cells holds great potential as a viable approach.

### 5.3 Cell replacement

Specific induction conditions can facilitate the differentiation of stem cells into contractile cardiomyocytes, ECs, and smooth muscle cells so that myocardial contractile function, vascular regeneration, and myocardial regeneration are enhanced, thereby improving cardiac function. Consequently, stem cell therapy represents a promising method for replenishing regenerative cells. Currently, induced pluripotent stem cells (iPSCs), mesenchymal stem cells (MSCs), cardiac stem cells (CSCs) and embryonic stem cells (ESCs) are among the primary types of stem cells employed for treating CVDs. Among these cells, the clinical application of ESCs is constrained by ethical considerations and the potential for immune rejection.

IPSCs are cells with self-renewal and pluripotent differentiation abilities obtained from autologous mature somatic cells after reprogramming. Since Takahashi et al. discovered iPSCs in 2006, their potential therapeutic effects on diseases, especially CVDs, have been explored (240). Animal experiments indicated that iPSCs could successfully differentiate into vascular ECs, cardiomyocytes and VSMCs. Furthermore, injection of iPSCs into ischemic myocardial tissue of rats has been shown to increase cardiac ejection fraction and reduce fibrosis (241, 242). Another study revealed that the integration of iPSC-derived cardiomyocytes, ECs, and VSMCs into the ischemic myocardium of pigs via intramyocardial microsphere transplantation enhanced the left ventricular ejection fraction, improved myocardial metabolism, and reduced the infarct size (243). In addition to the above animal experiments, Osaka University officially performed a phase I clinical trial of hiPSC-CM myocardial patches in January 2020 to assess their safety and potential efficacy in the hearts of patients with ischemic cardiomyopathy (237). The above evidence suggests that iPSC-CMs can be used as a new method for cardiac regenerative therapy.

MSCs are a subset of adult stem cells with the capacity to differentiate into mesodermal derivatives (chondrocytes, osteocytes, and adipocytes), have powerful multilineage differentiation potential and self-renewal ability, and have been widely used to alleviate ageing-related diseases. MSCs primarily treat ischemic CVDs through the following mechanisms (1): MSCs stimulate the proliferation and differentiation of cardiac cells, as well as angiogenesis (240) (2). MSCs promote cardiac repair and reduce myocardial apoptosis by secreting growth factors and exerting paracrine effects (244). Clinical trials of MSCs in CVD treatment are also more mature, multiple trials have been completed, and the expected results have been obtained.

In 2005, Hare et al. first used MSC transplantation to treat MI, and this study yielded crucial findings regarding the safety and effectiveness of allogeneic bone marrow stem cell applications (245). A clinical trial conducted in 2015 involved a controlled, multicenter randomized study of patients with chronic ischemic cardiomyopathy. The aim of this study was to evaluate the safety and efficacy of intramyocardial transplantation of allogeneic human MSCs derived from the umbilical cords of different individuals (246). In addition, Bartolucci et al. assessed the safety and effectiveness of administering intravenous infusions of MSCs derived from human umbilical cords to individuals diagnosed with chronic heart failure (247).

The above experiments demonstrated the safety of MSC transplantation as well as the efficacy of improving myocardial perfusion after MI. However, current clinical trials in CVD patients are still at a very early stage, and some potential risks associated with the systemic application of MSCs, such as embolism and inflammation, still exist. In addition, the potential differences in efficacy between MSCs from different sources must be circumvented and elucidated in future studies.

Whether CSCs can be used for the treatment of CVDs is controversial. In 2003, Beltrami et al. concluded that endogenous stem cells exist in the heart and that c-Kit^+^ cardiomyocytes cultured *in vitro*, enriched, and injected into necrotic cardiomyocyte areas were able to repair most necrotic areas and improve cardiac systolic function (248). The study had significant repercussions in academia, followed by the successive discovery of CSCs with different surface markers, and the locations and proportions of various CSC distributions have varied (249, 250).

However, the same approach was used by Jesty et al. (251) but did not replicate the findings of Beltrami et al. (248) that c-Kit^+^ CSCs can be converted into cardiomyocytes in the infarcted myocardium of adults. Van Berlo et al. (252) also questioned the role of CSCs in treating MI reported by Beltrami et al. In 2018, after an investigation by Harvard University and other relevant authorities, Beltrami et al. were suspected of fabricating data and paper fraud, which basically halted clinical trials of CSCs for the treatment of CVDs.

However, in some clinical trials, such as the SCIPIO trial (253) and the CADUCEUS trial (250), an intracoronary injection of endogenous CSCs has been observed to enhance the left ventricular ejection fraction, reduce the size of the myocardial infarct and the amount of scar tissue, and enhanced regional systolic function in patients with myocardial ischemia. Therefore, although CSCs with different surface markers cannot differentiate into cardiomyocytes, the paracrine effects of these cells can potentially enhance the movement, growth, specialization, and angiogenesis by cardiac stem cells within the body. Moreover, they can enhance the recruitment of endogenous CSCs, hinder cell apoptosis in the infarct region, inhibit myocardial remodeling, and thus improve cardiac function (254, 255). Given that adult cardiomyocytes still possess a relatively sluggish capacity for cell division, enhancing their ability to proliferate into cardiomyocytes and replace necrotic cardiomyocytes following myocardial ischemia could emerge as a prominent area of focus in future research.

### 5.4 Other factors

In addition to pharmacological interventions, increasing evidence suggests that modifiable lifestyle and environmental factors play an important role in modulating cardiac ageing.

Exercise has emerged as an effective strategy for the prevention and rehabilitation of CVDs. Exercise promotes mitochondrial biogenesis through AMPK regulation, increases cellular energy metabolism, and enhances functional reserve in the cardiovascular system. In addition, regular aerobic exercise can reduce oxidative stress in ECs and suppress aging-related inflammatory processes (256).

Dietary patterns significantly influence the ageing process. Both caloric restriction (CR) and intermittent fasting (IF) have been shown to delay aging through telomere lengthening and modulation of key signaling pathways including AMPK, PKB/AKT, and mTOR (257). Adherence to the EAT-Lancet dietary pattern - characterized by increased consumption of vegetables, fruits, whole grains, and nuts, along with reduced intake of animal-derived foods, red meat, added sugars, and saturated fats - has been associated with decelerated biological aging and extended life expectancy (258). This dietary approach provides abundant bioactive compounds such as omega-3 fatty acids, antioxidants (vitamin C, carotenoids, and polyphenols), zinc, and vitamin D, which exert multi-faceted anti-ageing effects. Its mechanisms of action mainly include enhancing innate and adaptive immune function, reducing oxidative stress damage, and improving cellular metabolic homeostasis, thereby effectively delaying aging-related inflammatory processes (259). The potential synergy between dietary interventions and senolytic therapies represents an emerging research frontier. Interestingly, β-hydroxybutyrate (β-HB) may serve as a crucial mediator connecting ketogenic diet, intermittent fasting (IF), and exercise with extended health span. The underlying mechanisms involve its anti-inflammatory properties, attenuation of vascular aging processes, and maintenance of immune homeostasis through CD8^+^T cell regulation (260).

Conversely, environmental toxins significantly accelerate aging processes through sustained genotoxic stress. Chronic exposure to airborne particulate matter (PM2.5) promotes DNA damage, micronuclei formation, and cGAS activation (261). Notably, smoking cessation represents a key lifestyle intervention that reduces inflammation and improves immune function (262).

## 6 Conclusions

With scientific and technological advancements and the evolution of society, the ageing population trend has become an inevitable social and medical problem in various countries around the world. Ageing research has experienced unprecedented momentum and potential in recent years. Previous findings indicate that the accumulation of senescent cells potentially plays a role in the progression of pathological states in different regions of the cardiovascular system. The rapid development of antiaging drugs and ageing intervention technologies will significantly benefit various aspects, such as human health, medical progress, and socioeconomic status. Despite these advancements, the comprehension of the specific molecular mechanisms underlying cardiovascular cell senescence remains limited, for example, how the ageing of a single cardiac cell type leads to a specific disease phenotype and how to screen highly selective markers for ageing cardiovascular cells (10). These conditions have hindered the development of effective methods to prevent or treat CVDs.

Investigational treatments are currently being explored with the goal of achieving overall suppression of senescence and/or clearance of senescent cells. Compared with senomorphics, which transiently reduce SASP levels, senolytics have rapidly become a reasonably effective and advantageous therapeutic strategy to prevent, delay, or reduce various age-related diseases and organ dysfunctions in recent years (263). Although multiple clinical trials on senolytic interventions are currently being conducted, none have targeted CVDs. In the future, more extensive randomized controlled trials must be conducted to accurately assess and ensure medication safety and treatment benefits and validate the preliminary results of early clinical trials. In addition, gaining a more comprehensive comprehension of the molecular mechanisms underlying immune response is imperative. Furthermore, the specific identification and targeting of ageing cardiovascular cells are crucial. Additionally, advancements in genetic, epigenetic, or metabolomic mechanisms related to cardiac cell senescence may provide enhanced personalized therapeutic options for individuals suffering from CVDs.
